# Integrating Selection Mapping With Genetic Mapping and Functional Genomics

**DOI:** 10.3389/fgene.2018.00603

**Published:** 2018-12-10

**Authors:** Martin Johnsson

**Affiliations:** ^1^The Roslin Institute and Royal (Dick) School of Veterinary Studies, The University of Edinburgh, Edinburgh, United Kingdom; ^2^Department of Animal Breeding and Genetics, Swedish University of Agricultural Sciences, Uppsala, Sweden

**Keywords:** selection mapping, genetic mapping, adaptation, selective sweep, population genomics

## Abstract

Genomic scans for signatures of selection allow us to, in principle, detect variants and genes that underlie recent adaptations. By combining selection mapping with genetic mapping of traits known to be relevant to adaptation, we can simultaneously investigate whether genes and variants show signals of recent selection and whether they impact traits that have likely been selected. There are three ways to integrate selection mapping with genetic mapping or functional genomics: (1) To use genetic mapping data from other populations as a form of genome annotation. (2) To perform experimental evolution or artificial selection to be able to study selected variants when they segregate, either by performing genetic mapping before selection or by crossing the selected individuals to some reference population. (3) To perform a comparative study of related populations facing different selection regimes. This short review discusses these different ways of integrating selection mapping with genetic mapping and functional genomics, with examples of how each has been done.

## Introduction

Genomic scans for signatures of selection allow us to, in principle, detect variants and genes that underlie recent adaptations. However, the results of selective sweep mapping and differentiation scans are not necessarily easy to interpret. Occasionally, scans may detect genes of known significance, such as known causative genes for monogenic traits [like the *BCDO2* yellow skin allele in the chicken (Rubin et al., 2010), pigmentation genes such as *MC1R*, *KIT*, and *MITF* (Rubin et al., 2012; Ramey et al., 2013; Qanbari et al., 2014), and double muscling alleles at *MSTN* (Bovine HapMap Consortium, 2009) in cattle], but in most cases, the genes and variants detected are of unknown phenotypic consequence. To better understand the results from genomic scans, one option is to combine them with genetic mapping of traits known to be relevant to adaptation and with genomic assays of gene function. In this way, we can simultaneously investigate whether genes and variants show signals of recent selection and whether they impact traits that have likely been selected. Integrating the two types of data allows phenotypic and functional data to corroborate selection mapping, and selection mapping to fine-map genetic mapping results in search of causative variants. This short review discusses different ways of integrating selection mapping with genetic mapping and functional genomics, with examples of how it has been done. The review will focus on animals and plants, but the concepts should be applicable to sexually reproducing organisms in general.

For the purpose of this article, “selection mapping” means any selective sweep or differentiation scan across the genome, and “genetic mapping” refers to any linkage or association study that connects genetic variants to traits. Both selection mapping and genetic mapping can be performed with a host of different statistical methods and use data from different genotyping and sequencing methods. Both can be performed genome-wide or targeted to a genomic region of particular interest. These differences may have important consequences for both the statistical properties and costs of the studies but will be abstracted away from most of the present discussion.

Selection mapping methods include those aimed at capturing classical “hard” sweeps, which occur through the fixation of one haplotype (Smith and Haigh, 1974), and “soft” sweeps (Hermisson and Pennings, 2005; Pennings and Hermisson, 2006a,b) where standing variation, migration, or recurrent mutation leads to fixation of a causative variant on the background of multiple haplotypes. It also includes methods to detect polygenic adaptation (Daub et al., 2013; Bourret et al., 2014; Foll et al., 2014) in the form of allele frequency shifts at many variants without fixation, analyses of time series polymorphism data (Illingworth et al., 2011), or studies that measure the association of population genetic parameters to environments in related populations (Joost et al., 2007; Coop et al., 2010; Villemereuil and Gaggiotti, 2015).

However, for the purpose of this discussion, it excludes methods that look for evidence for selection on a trait (Coop et al., 2010; Beissinger et al., 2018) with the help of polygenic scores or estimates from genome-wide regression. Nonetheless, the concepts are related, and the ideas of combining polymorphism and trait information in the same model and of avoiding thresholding genetic signals into quantitative trait locus peaks may be useful in future developments.

Genetic mapping and selection mapping both struggle when the genetic variants contributing to a trait have small effects but for different reasons. Genetic mapping studies need the association between markers and one or more causal variants to be strong enough to be detectable. Selection mapping studies need genetic variants to have experienced large allele frequency shifts. Modeling suggests that selection on a quantitative trait can either result in selective sweeps at a few large effect variants or subtle shifts at many small effect variants (Jain and Stephan, 2017a,b). Both cases can add up to large and rapid changes in phenotypes, and the important difference is the effect sizes of the variants relative to mutation rate.

For the purpose of this article, “functional genomics” refers to any genome-wide assay of gene function, such as gene expression, proteomics, chromatin immunoprecipitation sequencing, etc. It includes study designs that measure the average level of such molecular variables in a reference population, in functional genomics reference projects such as ENCODE (ENCODE Project, 2012), modENCODE (Celniker et al., 2009; Kudron et al., 2018), and gene expression atlas projects (Clark et al., 2017). It also includes genetical genomics, where the molecular variables are treated as quantitative traits and mapped with linkage mapping or genome-wide association methods (Jansen and Nap, 2001).

What we, as evolutionary geneticists, would like to do is quite simply to perform selection mapping and genetic mapping with the same genetic variants. The obvious problem with that is that if a causal variant has been fixed, there is no genetic variation left at that locus to map. Even if it has not been fixed, the minor allele frequency may be low, which complicates genetic mapping.

**Figure 1:**
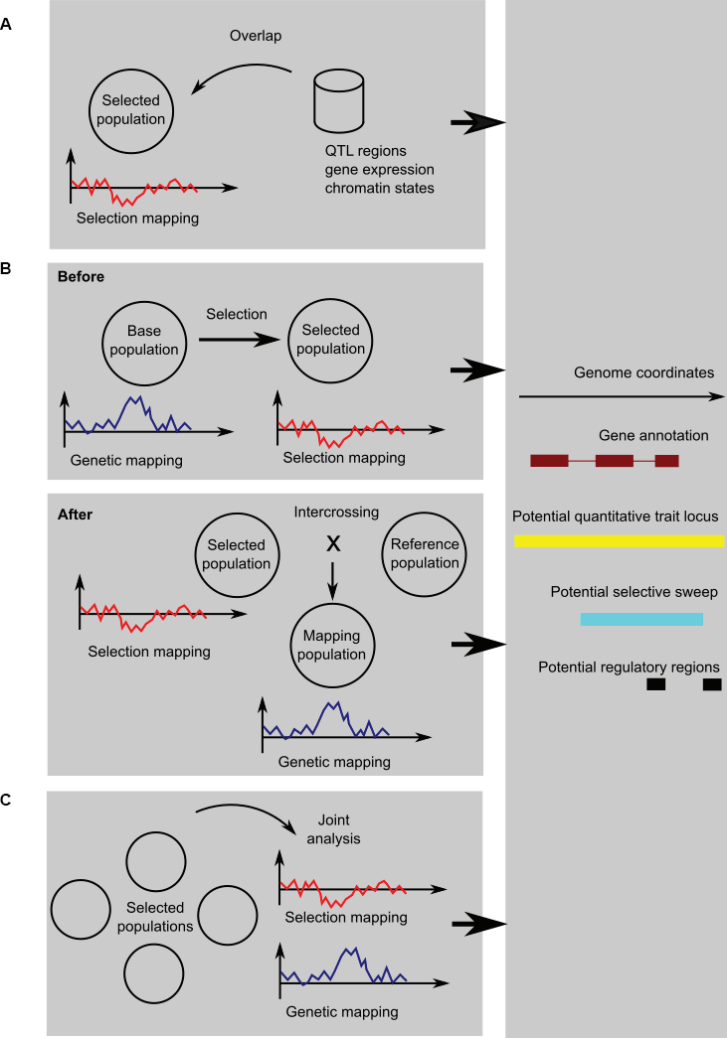
Schematic of the three kinds of designs: **(A)** using genetic mapping and functional genomics as annotation information; **(B)** genetic mapping before or after selection; and **(C)** comparative genetic and selection mapping using multiple populations. In the cartoon graphs, the x-axis represents coordinates along the genome, and the y-axis a test statistic for genetic mapping (for example, a LOD score from linkage mapping, a negative logarithm of the *p* from a genome-wide association study, etc.) or selection mapping (for example, change in allele frequency, Fst, pooled heterozygosity, or one of the haplotype sweep statistics). In all cases, the result is an integrated view of potentially selected, trait-associated, and functional regions, here shown as a genome-browser style view of a candidate region.

There are, broadly speaking, three ways to integrate selection mapping with genetic mapping or functional genomics (shown schematically in Figure 1). They each address the problem of fixed or low-frequency causative variants in different ways:

1. To use genetic mapping data from other populations as a form of genome annotation; the same applies to functional genomics or other genomic data, should they be available.
2. To perform experimental evolution or artificial selection and study selected variants when they segregate, either by performing genetic mapping before selection or by crossing the selected individuals to some reference population.
3. To perform a comparative study of related populations facing different selection regimes; even if a causative variant has been fixed in one population, it may be segregating in different populations, so that they can in effect serve as controls for each other.

## Genetic Mapping and Functional Genomics as Genome Annotation

The association of a gene or a genomic region with a trait in some population can be thought of as a fact about that gene or region. The logic is similar to how we might know that a gene encodes a protein kinase or that its product is located in the plasma membrane. Such information can be used to note overlaps between regions that show evidence of selection and regions associated with a trait or to perform enrichment analyses that consider all regions together. This is routine in selective sweep mapping studies of various of domestic animals [sheep (Moradi et al., 2012), pigs (Moon et al., 2015), pig (Wang et al., 2014), chicken (Gholami et al., 2015; Stainton et al., 2015), turkey (Aslam et al., 2014), cattle (Qanbari et al., 2010)], where the results are compared to quantitative trait loci from other studies or Animal QTLdb (Hu et al., 2015).

Such results are more or less convincing depending on the genetic distance between the population studied and the mapping population and the complexity of the trait. For example, an overlap between a known pigmentation gene, even one known only from homology with other species, and a sweep signal in a population known to have changed in pigmentation is compelling. On the other hand, an overlap between a sweep and a quantitative trait locus for a complex trait is weaker evidence because there are many quantitative trait loci, and each one covers many candidate genes.

Thus, overlapping selection mapping results with genetic mapping results is an attractive use of pre-existing data to interpret selection mapping. Has it helped derive new biological insights? The problem is that, except for single-gene major effects such as pigmentation genes, neither selective sweeps nor quantitative trait loci are usually resolved to the gene level, let alone to the variant level. Thus, we do not know whether the overlaps in question identify truly selected causative variants or not. In the case of enrichment analyses, it is not even clear how to test whether an observed enrichment represents a true target of selection or not.

The problems derive both from the fact that the information from genetic mapping, and to lesser extent functional genomics assays, is imprecise in terms of genomic resolution and that it may not reflect the genetic architecture in the selected population. When working with wild populations and non-model organisms, functional genomic data will most likely be derived from lab populations, or lifted from different species by sequence similarity (Nguyen et al., 2017; Naval-Sanchez et al., 2018). Genomic data derived from the population and its natural environment may be more precise, but at the same time much harder to get because of issues with accessibility, sample handling, and environmental noise.

There are additional problems related to enrichment testing. On the one hand, enrichment testing is appealing because it avoids the cherry-picking involved in finding overlaps with candidate genes and because it appears to provide a systematic way to treat long gene lists. However, simulation shows how enrichment testing in a selection mapping context is vulnerable to producing enrichment signals out of nothing (Pavlidis et al., 2012). Also, the common contingency table approaches overstate the evidence of enrichment because they test the null hypothesis of repeated sampling of gene lists (Goeman and Bühlmann, 2007; Khatri et al., 2012), which does not correspond to the uncertainties in real data.

Selection mapping methods can be possibly modified to incorporate annotation information in the same model, instead of performing overlaps and enrichment testing after the fact. There are methods for incorporating genome annotation (including functional genomics, gene annotation, and Gene Ontology terms) in genetic mapping and genomic prediction methods (Kichaev et al., 2014; Speed and Balding, 2014; Finucane et al., 2015; Edwards et al., 2016). Similarly, some models of polygenic adaptation already make use of pathway and ontology term annotation (Daub et al., 2013), and there are models of evolutionary constraint on annotation features that can integrate different annotation and functional genomics data (Gulko et al., 2015; Huang et al., 2017).

## Genetic Mapping Before or After Selection

To combine genetic mapping and selection mapping in the same population, one can either perform genetic mapping in the base population before applying selection, or do it afterwards by crossing a selected population to a reference population, which may be unselected or selected in the opposite direction. These designs lend themselves most naturally to experimental evolution and selection experiments but may also work in natural or domestic settings where time series data are available. As one might expect, it is beneficial if selection is replicated and if divergent selection is used (Kessner and Novembre, 2015).

Examples of genetic mapping applied before selection include studies where the base population for a selection experiment was started from a synthetic population. Evolve and resequence strategies, where genome-wide allele frequencies are measured before and after selection, are quite common in experimental evolution studies of model organisms, and some studies have combined them with genome-wide association studies, for example, to study courtship song in fruit flies (Turner and Miller, 2012; Turner et al., 2013). In this case, the base population was created by crossing the inbred lines used for the association study. The genome-wide association study, with a sample size of 168 genotypes, struggled to find significant hits, and the experimental evolution study on its own found thousands of putatively selected variants of unknown significance. Combined, they did support an enrichment of association with courtship song in differentiated regions, consistent with a polygenic architecture, and identified one candidate gene that was corroborated by a complementation test.

Another example of genetic mapping applied before selection, used in plant evolutionary biology is to plant a mapping population in a naturalistic setting (Ågren et al., 2013; Dittmar et al., 2014). Kerdaffrec et al. (2016) used this strategy to estimate selection on the *DOG1* gene, identified in a genome-wide association study for delayed germination in *Arabidopsis thaliana.* In this case, the study was targeted to a particular quantitative trait locus. They planted their mapping population in a naturalistic common garden and measured changes in haplotype frequencies over a summer of germination and growth. This means that they could measure the change in the frequency of the haplotype associated with delayed germination. The dormant haplotype increased as expected in this environment, consistent with the hypothesis of local adaptation.

An example of genetic mapping after selection comes from the Virginia chicken lines selected for body mass, which have been studied by selection mapping (Johansson et al., 2010; Pettersson et al., 2013; Lillie et al., 2017) and a series of quantitative trait locus mapping studies using intercrossing of the lines after selection (Jacobsson et al., 2005; Wahlberg et al., 2009; Sheng et al., 2015; Zan et al., 2017). Combining selection mapping and genetic mapping allowed researchers to test selective sweeps for association with the selected trait, which in this case is known by design. This revealed that about 10% of sweeps were associated with body weight, while the majority of them are likely to represent drift. It also helped pinpoint a likely novel mutation that appeared and fixed during the experiment. In summary, the response to selection in the Virginia chicken lines appears to be underpinned by both new and standing variations and by a smaller number of large-effect variants on a polygenic background.

When a study population is constructed by means of crossing, the biggest problem is obtaining the genomic resolution to separate linked variants. The population is designed to have causative variants segregating at high frequency; in the extreme case of a F_2_ cross of a selected population carrying a fixed causative variant with a reference population that are fixed for another allele, the frequency in the mapping population will be 0.5. However, the genomic resolution will be poor unless many generations of crossing or a large population size are used (Darvasi and Soller, 1995). A considerable effort may thus be required to set up the crosses. Getting at the causative variations requires laborious fine-mapping methods (Complex Trait Consortium, 2003) and is probably only possible in model organisms.

## The Comparative Design

The comparative design means sampling a series of populations and performing comparative selection mapping between them and joint genetic mapping of them collectively. It is an appealing approach for several reasons: it may not require artificial selection or crossing and thus promises naturalistic settings for the study. It also allows for studies of the repeatability and parallelism of evolution. Comparative designs are used in genetic mapping studies of local adaptation across panels of diverse populations and to detect local adaptation by association between population parameters and environmental variables measured on multiple populations.

Joint genetic mapping in structured populations is more challenging than mapping in a single well-mixed population, but it can be done with linear mixed models that take into account both relatedness and population structure (Yu et al., 2006; Zhao et al., 2007). If fixation would be completely parallel, causative variants would be lost, and uniquely fixed variants would be difficult to map because population identity and the causative variant would be confounded. However, given a quantitative trait of sufficient genetic complexity and populations facing different environments, the probability that the same allele will have swept to fixation in multiple populations should be small.

Examples include simultaneous comparative selection mapping by differentiation and across-breed genome-wide association studies in dogs (Vaysse et al., 2011; Long et al., 2013); studies of domestication and improvement in soy bean (Zhou et al., 2015); and studies using restriction site-associated sequencing in nonmodel birds (Chaves et al., 2016; Armstrong et al., 2018). Zhou et al. (2015) sequenced the genomes of domestic and wild soybean varieties and performed both selection mapping by a cross-population method and genome-wide association using a linear mixed model that used both kinship and principal components of genotypes to deal with population structure. By including both landraces and modern improved varieties, they were able to perform selection mapping separately for early domestication and later improvement and detect concordant loci between selection mapping and genome-wide association hits for several domestication- and improvement-related traits. Domesticated organisms seem likely to be amendable to this kind of study because of the strong divergence in known traits, the availability of diverse breeds and varieties, and the relative availability of genomic tools.

By virtue of large genome-wide association studies and population sequencing efforts, humans have become somewhat of a model organism for population genomics (see reviews by Akey (2009) and Scheinfeldt and Tishkoff (2013)). Some human studies combining selection mapping and genetic mapping would fit in the genome annotation category (for example, studies identifying a selective sweeps that overlap genes associated with Mendelian traits that may give hints about their function, such as albinism-associated genes in Voight et al. (2006)). However, the largest promise for the future lies in comparative studies of different human populations to identify the genetic basis of recent adaptation. For example, there is an overlap between signatures of selection in African populations with short, average height, and genome-wide association hits for height in non-African populations (Jarvis et al., 2012). To fully realize this promise, genome-wide association studies of the same traits in many human populations will be needed.

## Conclusion

In summary, all three designs have been used to combine selection mapping and genetic mapping in studies of adaptation. Looking forward, one might predict that:

1. Experimental evolution and selection experiments will lead the way, as will studies of model organisms and near-model organisms such as farm animals and crops. Gradually, and across the board, functional genomic data will become more available even for nonmodel organisms.
2. Statistical or machine learning models that include different kinds of genomic data as genome annotation in selection and genetic mapping will be a next logical development.
3. When the effects of individual loci are small, the sample sizes required for mapping will be huge, and adaptation may proceed by small shifts in allele frequencies at many variants. These problems will remain. However, with larger datasets and methods that make the best possible use of available data, the number of adaptive loci supported both by selection mapping and genetic mapping is likely to grow rapidly, even for complex traits.

## Author Contributions

The author confirms being the sole contributor of this work and has approved it for publication.

## Conflict of Interest Statement

The author declares that the research was conducted in the absence of any commercial or financial relationships that could be construed as a potential conflict of interest.
